# Global prevalence of prolonged grief disorder during the COVID-19 pandemic under standardized diagnostic frameworks: A systematic review and meta-analysis

**DOI:** 10.1017/S0033291726104541

**Published:** 2026-05-21

**Authors:** Shen Li, Lin Qiu, Yiyang Li, Xia Liu, Zining Luo, Jiaming Liu, Xuelei Ma

**Affiliations:** 1 Department of Biotherapy, West China Hospital and State Key Laboratory of Biotherapy, Sichuan University, Chengdu, China; 2 Department of Abdominal Oncology, West China Hospital of Sichuan University, Chengdu, China; 3 Department of Stomatology, North Sichuan Medical University, Nanchong, China; 4 Department of Urology, West China Hospital, Sichuan University, Chengdu, China

**Keywords:** COVID-19, prevalence, prolonged grief disorder

## Abstract

Prolonged grief disorder (PGD), recently classified in ICD-11 and DSM-5-TR, is characterized by persistent and functionally impairing grief lasting beyond 6–12 months. The COVID-19 pandemic was accompanied by widespread mortality, social isolation, disrupted mourning rituals, and social disconnection, raising concerns about a potentially high burden of PGD during the pandemic period. We conducted a systematic review and meta-analysis, following PRISMA guidelines and PROSPERO registration (CRD42023463720), to estimate PGD prevalence under standardized ICD-11 and DSM-5-TR diagnostic frameworks and to examine potential moderators during the COVID-19 pandemic. PubMed, EMBASE, and the Cochrane Library were searched from inception to October 2024. Eligible studies included adults who experienced bereavement during the pandemic and were assessed using validated PGD instruments (PG-13-R, ICG, BGQ). Random-effects models were applied to pool prevalence estimates, with subgroup and meta-regression analyses. Thirteen studies comprising 5,766 participants were included. The pooled prevalence of PGD during the pandemic period was 24% (95% CI: 13%–36%), with the highest estimates observed in China (43%, 95% CI: 33%–54%). In the overall pooled analysis, studies applying DSM-5-TR criteria yielded lower prevalence estimates than those using ICD-11 criteria (18% vs.26%, *p* = 0.41). Digital interventions showed no statistically significant pooled effects (Hedges’ *g* = −0.38, 95% CI: −0.90 to 0.14). The high and geographically heterogeneous prevalence of PGD observed during the COVID-19 pandemic underscores the need to strengthen mental health surveillance, standardized assessment, and service accessibility in large-scale public health emergencies, and provides important evidence to inform population-level interventions and resource allocation strategies.

## Introduction

Grief, as a multifaceted biopsychosocial response to bereavement, manifests as a dynamic process that includes emotional distress, cognitive restructuring, and functional adaptation. Existing studies confirm that 80%–90% of bereaved individuals gradually rebuild psychological adaptation through natural regulatory mechanisms, with emotional intensity and duration of emotion aligning with culturally accepted mourning practices (Lundorff, Bonanno, Johannsen, & O’Connor, 2020; Nielsen, Carlsen, Neergaard, Bidstrup, & Guldin, 2019). However, approximately 5%–10% of individuals develop pathological grief reactions, recently officially recognized as prolonged grief disorder (PGD) in the ICD-11 and DSM-5-TR, replacing non-standardized terms such as ‘complicated grief’ or ‘traumatic grief’ (Lundorff et al., 2020; Lundorff, Holmgren, Zachariae, Farver-Vestergaard, & O’Connor, 2017; Nielsen et al., 2019). Core diagnostic features include persistent (≥6–12 months) and intense grief reactions characterized by: (1) pathological yearning (≥1 symptom, e.g. persistent seeking of reminders of the deceased), (2) separation distress (≥1 symptom, e.g. identity confusion), and (3) functional impairment in social, occupational, or other domains, accompanied by additional comorbid symptoms (≥2 symptoms, e.g. emotional numbness or avoidance) (Prigerson, 2022). Notably, PGD is pathologically distinct from depressive disorders and post-traumatic stress disorder (PTSD), with its central theme revolving around loss, contrasting with the mood dysregulation in depression or traumatic re-experiencing typical of PTSD (Djelantik, Smid, Mroz, Kleber, & Boelen, 2020).

The global COVID-19 pandemic has precipitated an unprecedented public health crisis, resulting in millions of deaths and reshaping psychopathological trajectories of mental health through multifaceted trauma exposure mechanisms (Sokouti, Shafiee-Kandjani, Sokouti, & Sokouti, 2023). Beyond its acute mortality, the COVID-19 pandemic has generated substantial and uneven years lived with disability across global, regional, and local settings, highlighting profound health inequalities in its longer-term consequences. Such large-scale and disproportionate health losses provide an important population-level context for understanding the psychological sequelae of pandemic-related bereavement (Shan, Jin, Li, Yang, et al., 2025; Shan, Jin, Li, Zeng, et al., 2025). Initially, mass abnormal death events (e.g. in-hospital isolation deaths) and restricted funeral rituals disrupted the reparative function of traditional mourning practices. Additionally, social isolation policies diminished the buffering effects of support systems. Furthermore, pandemic-specific stressors increased the risk of comorbidities between pathological grief and health anxiety (Eisma, Boelen, & Lenferink, 2020; Vedder et al., 2022). Multinational epidemiological studies report a median PGD prevalence of 16.8% (IQR 9.5–22.3) among COVID-19, which is higher than many pre-pandemic prevalence estimates (9.8%, 95% Cl 6.8–14.0) (Lundorff et al., 2017). However, the inconsistencies in reported prevalence and impact estimates during the COVID-19 pandemic underline the need for high-quality evidence synthesis to address these uncertainties.

This meta-analysis aims to comprehensively evaluate the prevalence of PGD in the context of the COVID-19 pandemic. Given the historical heterogeneity in diagnostic criteria before PGD’s formal recognition, this study will exclusively include research utilizing ICD-11 and DSM-5-TR standardized assessment tools to enhance diagnostic accuracy. Our objectives are to provide robust prevalence estimates, identify potential moderators of risk, and inform evidence-based mental health support strategies during global public health crises.

## Methods

### Search strategy and inclusion criteria

This systematic review and meta-analysis strictly followed PRISMA guidelines and was registered on PROSPERO (CRD42023463720) (Moher, Liberati, Tetzlaff, Altman, & Group, 2009; Page et al., 2021). A comprehensive search was conducted in PubMed, EMBASE, and the Cochrane Library from database inception to 21/10/2024, employing a language bias mitigation strategy that allowed inclusion of non-English publications after professional translation. Data were first extracted on 24/10/2024. The search strategy used MeSH terms (COVID-19 and Prolonged Grief Disorder) combined with Boolean operators (as detailed in the Supplementary Information). Inclusion criteria required studies to: (1) involve adults (≥18 years) bereaved of spouses, blood relatives, or close social relationships during the COVID-19 pandemic; (2) assess PGD using validated tools such as the Revised Prolonged Grief Disorder Scale (PG-13-R, cut-off >30), Inventory of Complicated Grief (ICG, cut-off >25), or Brief Grief Questionnaire (BGQ, cut-off >4), aligned with diagnostic duration thresholds in ICD-11 (≥6 months) or DSM-5-TR (≥12 months); and (3) include cohort studies or cross-sectional designs. Exclusion criteria comprised studies mixing non-COVID-19-related bereavement cohorts, samples with comorbid severe mental disorders (e.g. schizophrenia spectrum disorders [ICD-10 F20-F29], major depressive disorders [F32-F33]), structured psychological interventions (e.g. CBT, EMDR), non-original research (e.g. case reports, conference abstracts), or gray literature lacking full- access. Studies reporting additional major stressors or trauma exposures were excluded to minimize confounding.

Two independent researchers (LS and QL) screened titles and abstracts, followed by full-text review. Data extraction covered study characteristics (title, first author, year), design, population demographics, PGD assessment tools and thresholds, prevalence estimates (with 95% confidence intervals), and intervention details (for interventional studies). Discrepancies were resolved through third-party arbitration (LX).

### Data analysis

Single-arm meta-analyses were conducted to synthesize PGD prevalence rates, reported as pooled percentages with 95% confidence intervals. To stabilize variances and accommodate proportions close to 0 or 1, raw prevalence rates were transformed using the Freeman–Tukey double-arcsine transformation before pooling (Barendregt, Doi, Lee, Norman, & Vos, 2013). The DerSimonian–Laird random-effects model and inverse-variance weighting were applied to account for heterogeneity (Higgins, Thompson, Deeks, & Altman, 2003). Heterogeneity was quantified using the *I* ^2^ statistic (with thresholds: ≤25% = low, 25–75% = moderate, >75% = high), interpreted alongside prediction intervals and clinical context due to limitations in single-rate analyses. Publication bias was assessed via visual inspection of funnel plot symmetry and Egger’s regression, and adjusted using trim-and-fill analysis (Egger, Davey Smith, Schneider, & Minder, 1997). Results were visualized through forest plots, where box sizes reflected study weights and diamond markers indicated pooled effect estimates. Subgroup analyses stratified findings by country, assessment tool, and study design to identify heterogeneity sources. Meta-regression explored associations between PGD prevalence and covariates. For studies evaluating digital interventions, Hedges’ *g* (adjusted for small-sample bias) was calculated as the standardized mean difference and analyzed via random-effects models. Missing means and standard deviations were estimated from medians and interquartile ranges, with results depicted in forest plots.

### Bias and quality assessment

Risk of bias was independently evaluated by two reviewers (LS, QL) using the Newcastle-Ottawa Scale (NOS), assessing domains such as sample representativeness, measurement validity, and confounder control (high quality defined as NOS score ≥ 7) (Peters, Sutton, Jones, Abrams, & Rushton, 2006). Evidence certainty was graded via the GRADE framework, incorporating adjustments for methodological limitations. Disagreements during quality assessment were resolved by a third reviewer (LJM) (Guyatt et al., 2011; Stang, 2010).

## Results

### Characteristics of included studies

**Figure 1.**
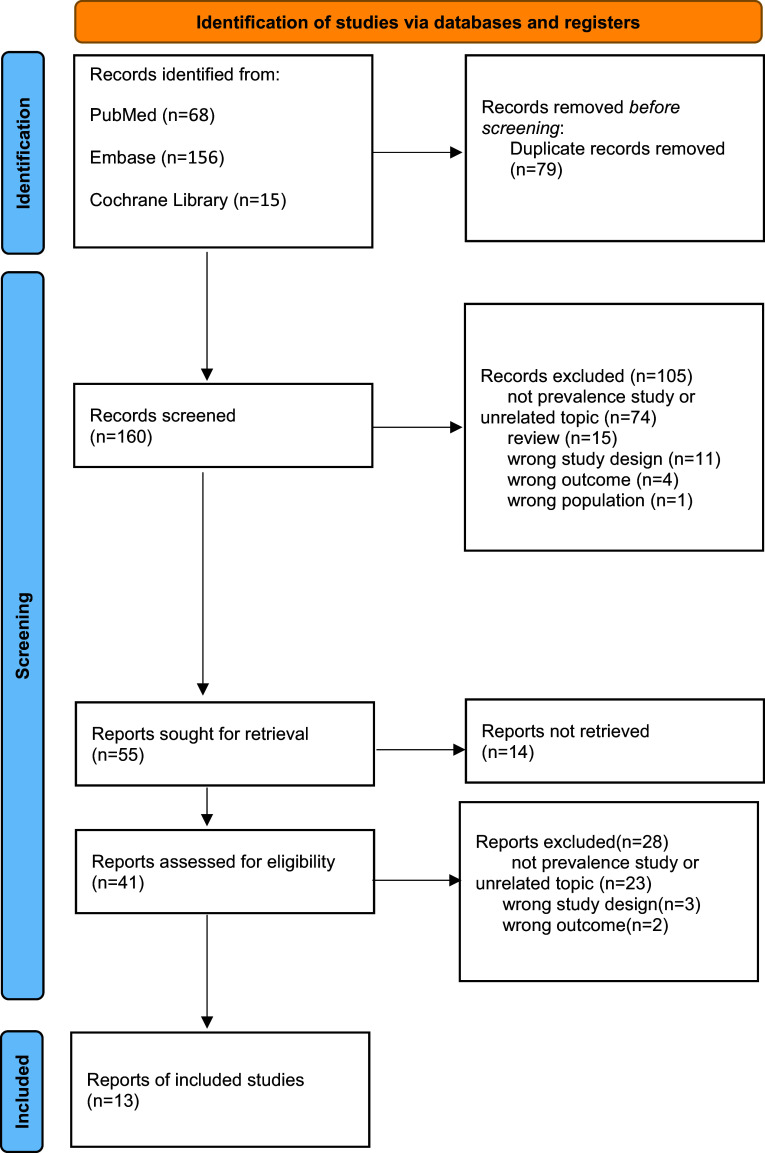
Flow diagram of study selection. Definitions of exclusion criteria: (1) Wrong study design: Studies not using cross-sectional or cohort designs (e.g. case reports, reviews, experimental studies without observational data on PGD prevalence); (2) Wrong outcome: Studies not reporting PGD-related outcomes (e.g. only reporting general grief symptoms without PGD-specific assessments); (3) Wrong population: Studies not focusing on COVID-19-bereaved populations (e.g. studies on bereavement due to other causes, non-bereaved populations).

A total of 13 studies from Iran, the US, Canada, Australia, Brazil, the UK, and China were included in this systematic review and meta-analysis. The age range of participants varied significantly, with a minimum mean age of 19.29 years and a maximum of 70.32 years, reflecting the distribution of PGD across age groups. Diagnostic criteria were based on DSM-5 (7 studies) and ICD-11 (6 studies). Gender distribution exhibited regional variations, with the male proportion ranging from 11.0% to 55.5% (Figure 1; Table 1).

**Table 1.** The characteristics of the included study (Aliyaki et al., 2024; Bedford, Trotter, Potter, & Schmidt, 2023; Downar et al., 2022; Harrop et al., 2023; Lapenskie et al., 2024; Lobb et al., 2024; Lucena et al., 2024; Rodriguez-Villar et al., 2024; Schneider et al., 2023; Shevlin et al., 2023a; Stahl et al., 2024; Tang & Xiang, 2021; Tang, Yu, Chen, Fan, & Eisma, 2021)

Author (year)	Research type	Country	Number (total)	Number (diagnosed with PGD)	Age (mean ± SD or median (IQR)	Gender (male percentage)	Diagnostic criteria	Scale	Measure	Population	Time	COVID implementation period	Recruitment method	Recruitment source
Aliyaki et al., 2024	Cross-sectional study	Iran	203	80	38.72(11.75)	39.2	DSM–5-TR	PG–13-R	≥30	general	> 6 months	Mar 2021–Jan 2023 (death period); Jan – May 2023 (data collection period)	Telephone screening + online survey (Porsline platform) distributed via Telegram/WhatsApp App; convenience sampling based on hospital medical records	First-degree relatives (18–65 years old) of decedents at Masih Daneshvari Hospital, Tehran, Iran; bereaved for 6–24 months; no severe psychiatric disorders; non-traumatic death
Stahl et al., 2024	Cohort study	America	20	8	70.32 (5.78)	15	DSM–5-TR	ICG	≥30	Elderly (≥60)	> 6 months	Jan 2020–Dec 2022 (enrollment period); assessments at 6, 12, and 15 months post-loss	Virtual recruitment for RCT (WELL study); electronic consent via REDCap; online surveys (Inventory of Complicated Grief) administered at baseline and 3-month intervals up to 15 months	Older adults (≥60 years old) in Pittsburgh, PA, USA; bereaved spouses (within 12 months of loss); at risk for depression (HRSD ≥9); excluded severe psychiatric disorders, dementia, or acute suicide risk
											12 months			
											15 months			
Lapenskie et al., 2024	Cohort study	Canada	111	32	58.2(15.0)	33.3	ICD–11	ICG	>25	general	> 6 months	Nov 2019–Aug 2020 (death period); 12–18 months post-death (follow-up assessment)	Telephone contact with initial cohort; verbal informed consent; Inventory of Complicated Grief (ICG) administered via phone; prospective matched cohort design	Family members (next-of-kin) of decedents in 3 acute care hospitals in Ottawa, Canada; bereaved 12–18 months prior; matched groups: COVID–19 death, non-COVID death (pandemic wave 1), and pre-COVID death
Lobb et al., 2024	Cross-sectional study	Australian	744	120	56.1(11.5)	6.3	ICD–11	PG–13-R	≥30	general	> 12 months	Jan 2020–Feb 2022 (death period); ≥2 months post-loss (survey participation)	Online survey via REDCap; advertised via social media (Facebook/Instagram), newsletters, websites, online fora, and bereavement services; no incentives offered	Australian adults (≥18 years); bereaved carers/family/close friends of persons who received palliative care and died at home or in hospital during the pandemic; English-speaking; excluded if <2 months since death
Lucena et al., 2024	Cross-sectional study	Brazil	44	5	41.3(12.4)	16	ICD–11	PG–13	≥30	general	> 6 months	Apr 2021–Jun 2021	Online survey (Google Forms) via social media, emails, and snowball sampling; no incentives	Brazilian adults (≥18 years old) bereaved due to COVID–19; with internet access and reading ability
Shevlin et al., 2023b	Cohort study	UK	1763	41 178	54.61 (14.24)	50	ICD–11	IPGDS	strict	general	> 6 months	Mar 2021–Apr 2021 (Wave 5 of C19PRC-UK); bereavement occurred ≥6 months prior to survey	Quota sampling via Qualtrics; recontact of previous participants from the C19PRC-UK study (Waves 1–4)	Bereaved adults (≥18 years old) from the UK C19PRC-UK longitudinal cohort; bereaved for ≥6 months
									moderate					
Schneider et al., 2023	Cross-sectional study	American	196	18	19.29(2.08)	31.1	DSM–5-TR	PG–13-R	≥30	youngster	> 12 months	Mar 2020–Jan 2021 (data collection); ≥6 months post-loss	Online survey via Qualtrics; convenience sampling distributed via social media, university listservs, and partner organizations; no incentives offered	US young adults aged 18–29 who experienced the death of a close family member or friend during the pandemic; bereaved for ≥1 year
Shevlin et al., 2023a	Cohort study	UK	1777	43	60.11(8)	50.1	ICD–11	IPGDS	strict	general	> 6 months	Mar 2021 (data collection); bereavement >6 months before survey	Online survey via Qualtrics; longitudinal re-contact of existing cohort (C19PRC-UK Wave 5); quota sampling for national representativity	UK adults (≥18 years) from a nationally representative panel who reported the death of someone close >6 months prior; bereaved for ≥6 months
				140					moderate					
Harrop et al., 2023	Cohort study	UK	383	167	51(13)	12	Not specified(TGI-SR cut-off ≥54 Indicate of PCBD/PGD)	TGI-SR	≥54	general	> 8 months	Aug 2020–Jan 2021(data collection) and follow-ups at c. 8, 13, and 25 months post-bereavement	Online survey (JISC Online Surveys) disseminated via social media, mainstream media, and voluntary sector/bereavement support organizations; hard-copy postal surveys available on request	UK adults (≥18 years) who were family members or close friends bereaved between 16 March 2020 and 2 January 2021
			295	102	51(13)	11					> 13 months			
			185	53	51(12)	14					> 25 months			
Downar et al., 2022	Cohort study	Canada	76	23	58.4 (14.7)	36.8	DSM-IV	ICG-R	>25	general	> 6 months	Nov 2019–Aug 2020 (death period); family members contacted >6 months post-loss	Phone contact with next-of-kin identified from hospital medical records; additional family members enrolled upon spontaneous request	Family members of patients who died in acute care hospitals in Ottawa, Canada between Nov 1, 2019 and Aug 31, 2020 (including pre-pandemic, non-COVID pandemic, and COVID–19 deaths)
Tang et al., 2021	Cross-sectional study	China	188	71	32.73 (9.31)	55.5	ICD–11	IPGDS	strict	general	> 6 months	Jan 2020–Oct 2020 (death period); Sep 2020–Oct 2020 (data collection)	Online survey distributed via social network websites (Baidu, WeiBo) and mobile applications (WeChat)	Chinese adults (≥18 years) who lost a close person due to COVID–19; convenience sample recruited through online platforms
				55			DSM–5	TGI - SR	≥54					
Tang et al., 2021	Cross-sectional study	China	188	92	32.73 (9.31)	56	ICD–11	IPGDS	≥ 42.5	general	> 6 months	Sep 2020–Oct 2020 (data collection)	Online survey distributed via social network websites (Baidu, WeiBo) and mobile applications (WeChat)	Chinese adults (≥18 years) bereaved due to COVID–19; recruited via online advertisements on social media platforms
Rodriguez-Villar et al., 2024	Cross-sectional study	UK	73	25	66(58–73)	71.2	DSM–5	PG–13-R	>30	general	> 12 months	Mar 2020–Dec 2021 (death period); Jul 2021–Nov 2021(data collection)	Telephone interviews conducted by trained interviewers; participants identified from medical records and contacted via phone	Next of kin of patients who died of COVID–19 in ICUs in South-East London (King’s College Hospital NHS Foundation Trust and Princess Royal University Hospital); recruited through hospital medical records

### PGD prevalence results

**Figure 2.**
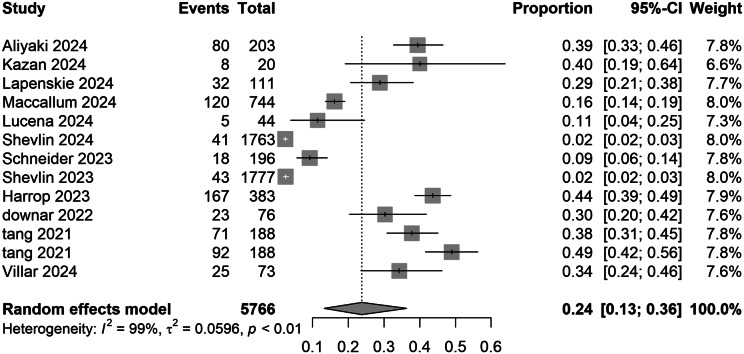
Pooled meta-analysis prevalence rates for COVID-19-related PGD in strict criteria.

Thirteen studies comprising 5,766 participants were included in the analysis of COVID-19-related PGD prevalence. The IPGDS defined diagnostic criteria as follows: score ≥ 4 (indicating ‘often’ or ‘always’) for strict criteria and ≥ 3 for lenient criteria. Two studies by Shevlin et al. (2023a, 2023b) were separately analyzed under these criteria. Under strict criteria, the random-effects model yielded a pooled proportion of 0.24 (95% CI: 0.13–0.36), with significant variability across studies (range: 0.02–0.49) and high heterogeneity ( *I* ^2^ = 99%). Lenient criteria produced a higher pooled proportion of 0.26 (95% CI: 0.18–0.35), with individual estimates ranging from 0.08 to 0.49 (Supplementary Information). Despite minor numerical differences, overlapping confidence intervals between criteria indicated no substantial impact of IPGDS threshold adjustments on overall estimates (Figure 2).

### Subgroup and meta-regression analyses

In country subgroups, Canada reported higher prevalence estimates than the UK (0.29 [0.23–0.36] vs. 0.16 [0.03–0.35]), while Chinese samples showed the highest prevalence (0.43, 95% CI: 0.33–0.54). A formal test for subgroup differences indicated evidence of regional variation (*Q*_between_ χ^2^ = 71.87, df = 6, *p* < 0.01). Overall heterogeneity across studies was high (*I* ^2^ = 98.2%), underscoring substantial between-study variability. Limited sample sizes (*n* ≤ 2) in regions such as the United States and Australia warrant cautious interpretation. Prevalence estimates did not differ materially between cross-sectional (0.27 [0.16–0.40]) and cohort studies (0.20 [0.07–0.37]) (*p* = 0.51), suggesting minimal design-related variability (Table 2).

**Table 2.** Pooled estimates of PGD prevalence during the COVID-19 pandemic and intervention efficacy

Analysis category	Diagnostic framework	Assessment tool	centerDuration threshold	Pooled proportion (95% CI)	Other effect size/test statistic	*p* Value/*κ* value	Sample size (*n*)
Overall pooled prevalence	DSM–5-TR	–	No restriction	18% (9%–29%)	–	0.41	5,766
Overall pooled prevalence	ICD–11	–	No restriction	26% (17%–35%)	–	–	5,766
Subgroup: by duration threshold	DSM–5-TR	–	≥12 months	52% (44%–60%)	χ^2^ = 18.3	<0.001	2,842
Subgroup: by duration threshold	ICD–11	–	≥6 months	76% (68%–84%)	–	–	2,842
Subgroup: aligned duration	ICD–11 (Aligned to DSM–5-TR)	–	≥12 months	54% (46%–62%)	–	κ = 0.91	2,842
Subgroup: by country	Combined	–	No restriction	China: 43% (33%–54%)	–	<0.01	376
				Canada: 29% (23%–36%)	–	–	187
				UK: 16% (3%–35%)	–	–	3,996
Subgroup: by assessment tool	Combined	TGI-SR	No restriction	44% (38%–49%)	–	<0.01	383
		PG–13/PG–13-R	No restriction	22% (8%–42%)	–	–	516
		ICG/ICG-R	No restriction	26% (16%–38%)	–	–	951
		IPGDS	No restriction	18% (5%–36%)	–	–	3,916
Digital intervention efficacy	Combined	–	–	–	Hedges’ *g* = −0.38	Non-significant	426

Assessment tools exhibited dose-dependent effects: studies using the TGI-SR reported the highest prevalence (0.44 [0.38–0.49]), significantly exceeding rates derived from PG-13 (0.22 [0.08–0.42]), ICG (0.26 [0.16–0.38]), and IPGDS (0.18 [0.05–0.36]) (*p* < 0.01). Diagnostic duration comparisons showed no significant difference between ICD-11’s 6-month criterion (0.26 [0.12–0.42]) and DSM-5-TR’s 12-month standard (0.18 [0.09–0.29]) (*p* = 0.19).

**Figure 3.**
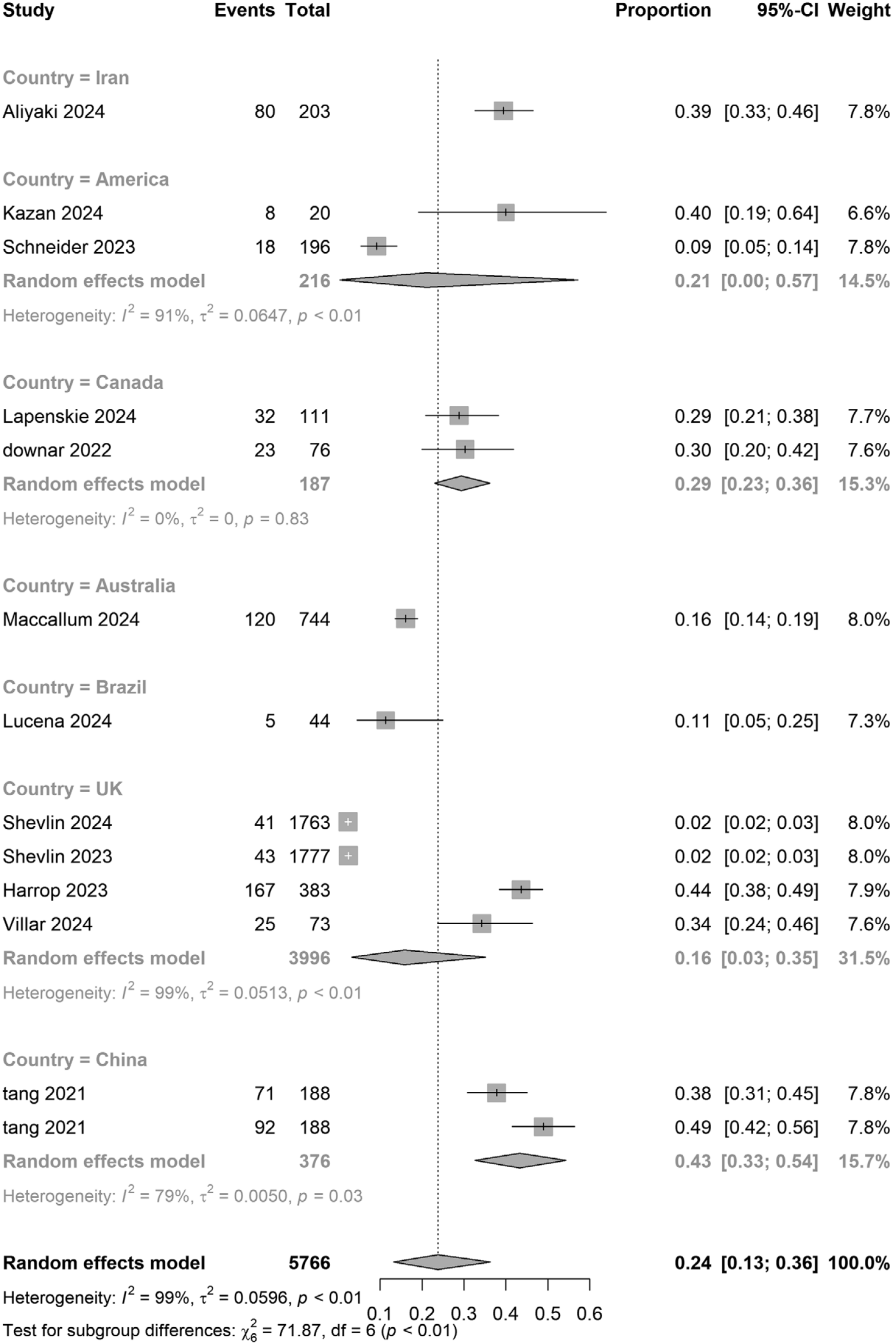
Pooled meta-analysis proportion for COVID-19-related PGD in national subgroups under strict criteria.

**Figure 4.**
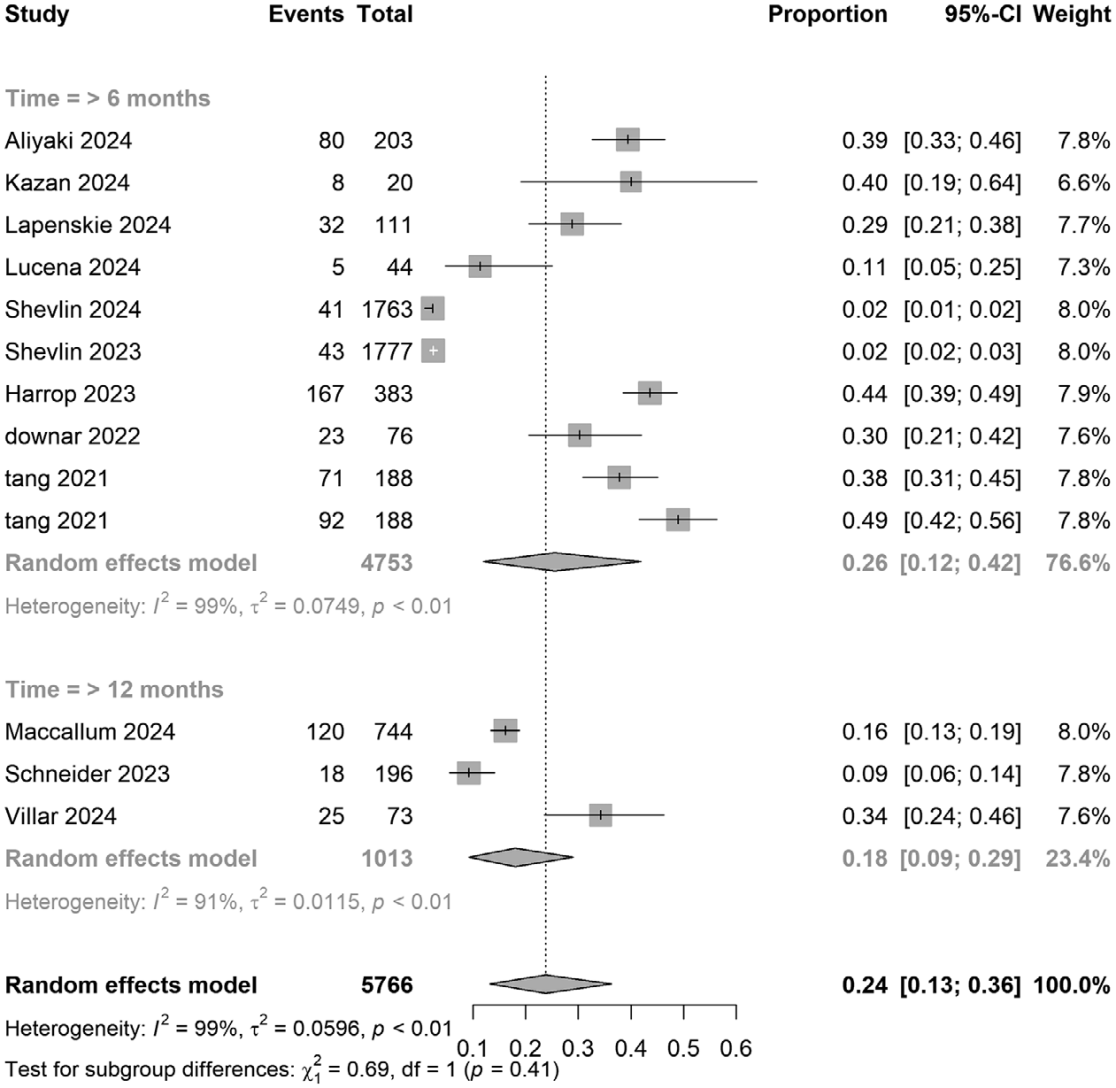
Pooled meta-analysis proportion for COVID-19-related PGD in follow-up time subgroups under strict criteria.

**Figure 5.**
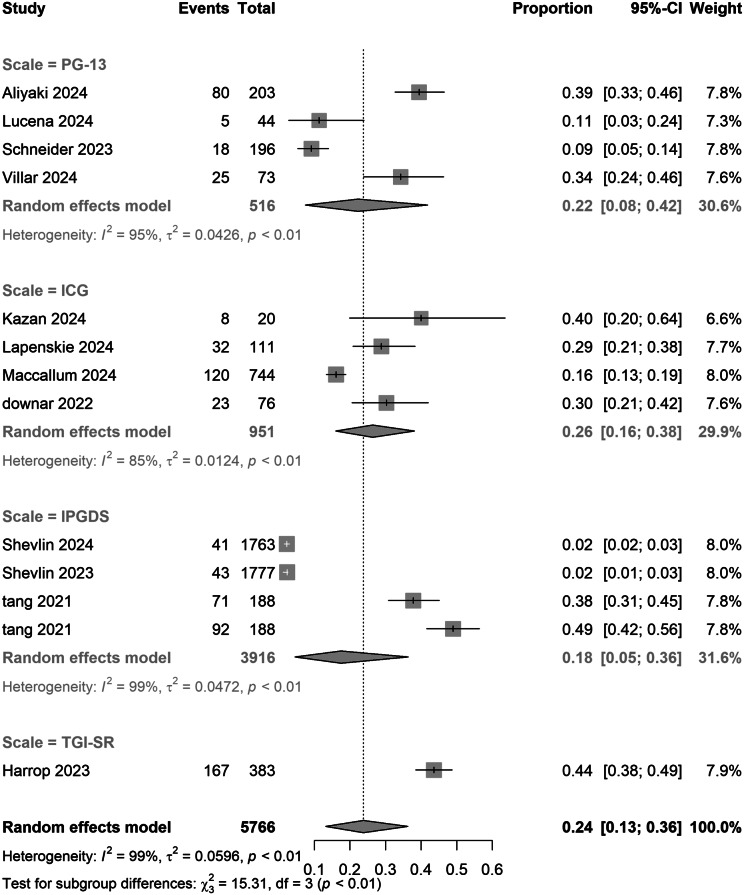
Pooled meta-analysis proportion for COVID-19-related PGD in scale subgroups under strict criteria.

**Figure 6.**
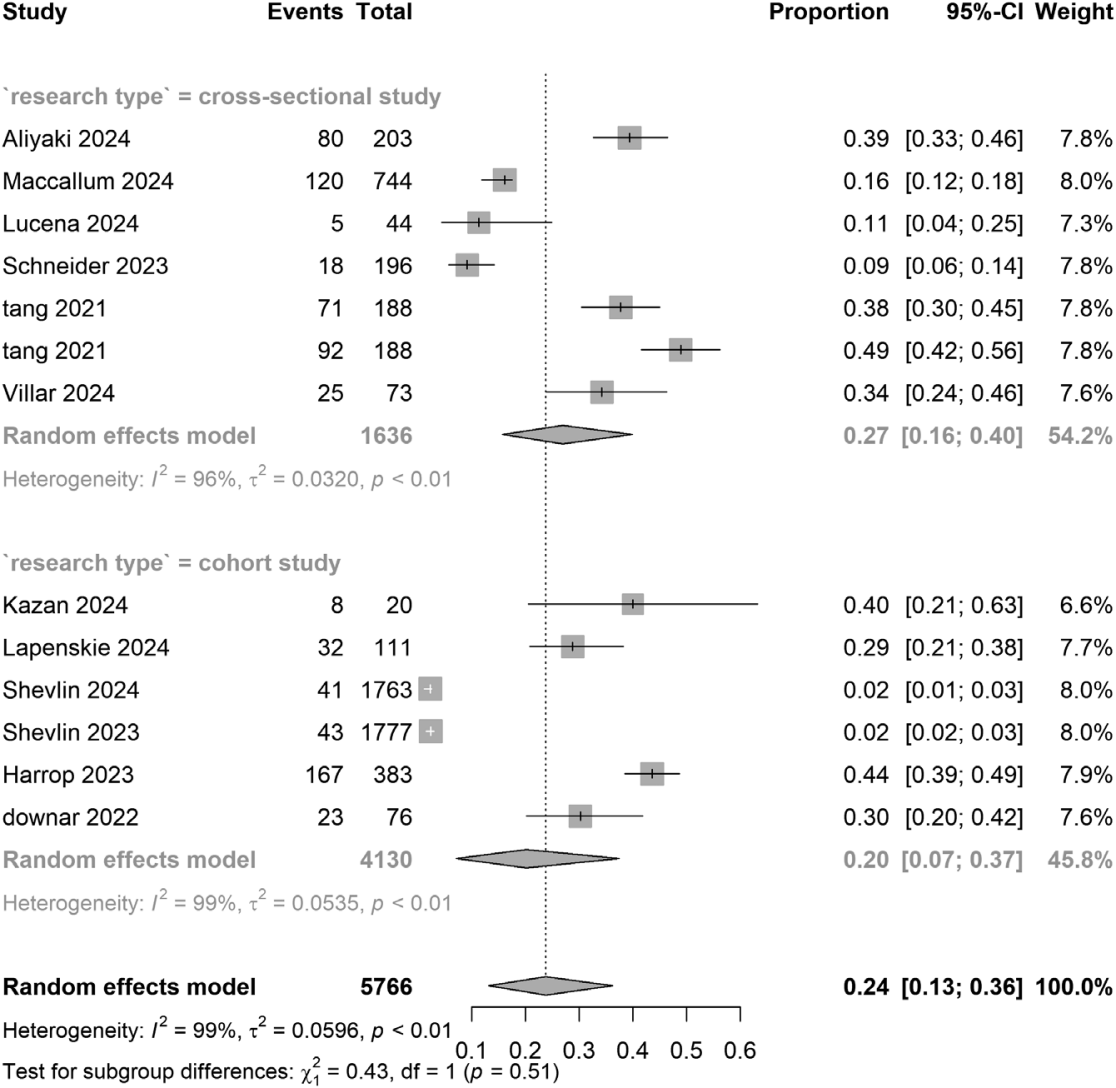
Pooled meta-analysis proportion for COVID-19-related PGD in study design subgroups under strict criteria.

Meta-regression revealed no statistically significant associations of PGD prevalence with mean age (*β* = −0.0008, *p* = 0.81) or female proportion (*β* = 0.0007, *p* = 0.78). Sensitivity analyses confirmed the robustness of subgroup findings across strict and lenient diagnostic thresholds (Supplementary Information; Figures 3– 6).

### Efficacy of digital PGD interventions

Digital interventions utilizing online psychotherapy and mobile applications were aimed at alleviating grief symptoms. The pooled Hedges’ *g* of −0.38 (95% CI: −0.90 to 0.14) under random-effects models did not reach statistical significance but suggested potential clinical utility. The secondary analysis of digital interventions for PGD, including detailed methods, risk of bias assessment, and results, is available in the Supplementary Material (Supplementary Information S3), as this analysis was not pre-specified in the original study objectives.

### Risk of bias and GRADE ratings

The GRADE framework rated the evidence certainty as very low, downgraded due to high heterogeneity and limited generalizability. Using the NOS, study quality scores ranged from 6 to 9 (out of 9), with seven studies achieving a total score of nine (indicating high quality). These studies demonstrated robust methodology in participant selection, comparability, and outcome assessment (Supplementary Information).

## Discussion

The COVID-19 pandemic has triggered a significant increase in mortality rates, forcing individuals to confront bereavement amidst concurrent pandemic-related stressors. Existing research has documented a surge in psychological challenges such as anxiety and depression during this period. Additionally, disruptions to normative grief processes, caused by restrictions on end-of-life interactions and limitations on funeral rituals, have amplified the risk of developing prolonged grief responses, which can severely impair daily functioning. This underscores the importance of investigating PGD among pandemic-bereaved populations. Although recent studies have explored PGD prevalence and risk factors in various contexts, no systematic review or meta-analysis has examined its epidemiological shifts during large-scale public health emergencies like COVID-19. This study represents the first comprehensive synthesis of PGD prevalence and its determinants across 13 studies involving 5,766 participants in this distinctive context.

As a result of the pandemic, bereavement experiences have been fundamentally altered due to increased mortality rates and fragmented social support systems. Our meta-analysis revealed a pooled PGD proportion of 24% (95% CI: 13–36%) based on strict diagnostic criteria, exceeding most prevalence ranges reported in pre-pandemic works. Using lenient criteria, the estimated prevalence rose slightly 26% (18%–35%). Using Lenferink, van den Munckhof, de Keijser, and Boelen’s (2021) dual-threshold framework, we found a marginal difference of 2% between the ‘moderate’ and ‘strict’ IPGDS criteria as defined by ICD-11, suggesting potential insensitivity in the calibration of diagnostic tools (Eisma et al., 2020; Lenferink et al., 2021). These findings highlight the impact of structural traumas, e.g. restricted access at the deathbed and disruptions to traditional mourning practices, which may prolong grief pathology beyond typical acute stress responses.

Notably, geographical subgroup analysis revealed an exceptionally high PGD prevalence of 43% in Chinese samples, sharply contrasting with rates of 29% in Canada and 16% in the United Kingdom. This divergence from pre-pandemic cross-cultural patterns may reflect the psychosocial impacts of China’s ‘dynamic zero-COVID’ policy, which intensified social isolation and grief-related loneliness through prolonged lockdown measures. Moreover, regional heterogeneity was statistically significant (*p* < 0.01), whereas study design (cross-sectional vs. cohort) showed no group differences (*p* = 0.51), implying universal risk factors among COVID-19-bereaved populations. Sensitivity analyses confirmed the robustness of subgroup results across diagnostic thresholds. However, data gaps in low-income regions, such as Africa and South Asia, and a high-income-country bias (78% of studies) limit global generalizability, necessitating multinational validation (Aliyaki et al., 2024; Lucena et al., 2024). Beyond these immediate epidemiological findings, the high burden of PGD observed may suggest potential long-term neurocognitive risks. Given that prolonged grief is a chronic stressor linked to cognitive decline precursors, and considering Shan et al.’s findings on COVID-19-related dementia risk, bereaved individuals might face a ‘dual hit’ of viral and psychosocial stressors. This synergy could imply that high PGD prevalence may accelerate cognitive decline, warranting consideration of PGD monitoring as a strategy to mitigate future neurocognitive disorders (Shan, Wang, Crawford, & Holland, 2024).

Methodological heterogeneity in PGD research, resulting from divergent diagnostic criteria, compromises the comparability of results. For instance, DSM-5-TR, which establishes a 12-month duration threshold, yielded a PGD prevalence of 52% (95% CI: 44%–60%), significantly lower than ICD-11’s 76% prevalence with a 6-month threshold (χ^2^ = 18.3, *p* < 0.001), indicating moderate diagnostic agreement (*κ* = 0.51). This subgroup result differs from the overall pooled prevalence reported in the Abstract (DSM-5-TR: 18% vs ICD-11: 26%, *p* = 0.41), due to heterogeneity in duration thresholds across included studies. When aligning the ICD-11’s duration criterion to 12 months, concordance improved markedly (*κ* = 0.91), highlighting the critical impact of threshold duration on diagnostic validity. This may suggest that the stricter symptom requirements of DSM-5-TR become more distinguishable from ICD-11 only after the acute grief phase has passed. Additionally, the selection of assessment tools influenced prevalence estimates: studies employing the TGI-SR reported the highest prevalence (44%, 95% CI: 38–49%), compared to those using the PG-13-R (22%, 8–42%) (SMD = 1.23, *p* = 0.008). Although no significant difference in prevalence was observed between 6- and 12-month follow-ups (26% vs. 18%, *p* = 0.19), the limited number of studies and potential regional bias necessitate cautious interpretation.

Subgroup and meta-regression analyses further examined demographic influences on PGD prevalence. The disparities observed between Western countries (Canada: 29%, UK: 22%) and Eastern countries (China: 43%) may reflect variations in cultural contexts, support systems, and pandemic policies. Notably, China’s elevated PGD rate contradicts pre-pandemic trends, potentially linked to strict isolation measures that exacerbate grief-related isolation. Both cross-sectional (27%) and cohort studies (20%) displayed comparable prevalence (*p* = 0.51), suggesting minimal design-related biases. Meta-regression analyses revealed no significant associations with mean age (*β* = −0.0008, *p* = 0.81) or the proportion of female participants (*β* = 0.0007, *p* = 0.78). Sensitivity analyses confirmed the robustness of subgroup findings across diagnostic thresholds (Supplementary Information).

The formalization of PGD in DSM-5-TR and ICD-11 introduced critical diagnostic divergences. The DSM-5-TR employs an algorithmic approach that prioritizes specificity (core criteria plus at least seven symptoms), while ICD-11 uses a typological method that emphasizes qualitative grief features. Although the ICD-11’s simplicity enhances its applicability across diverse cultural contexts, its lower symptom threshold poses a risk of overdiagnosis compared to prior criteria (e.g. PCBD). Adjusting the ICD-11’s duration criterion to 12 months significantly improved alignment with the DSM-5-TR (*κ* = 0.91, up from *κ* = 0.51), emphasizing the critical role of duration in diagnostic accuracy. This aligns with our subgroup analysis, where DSM-5-TR (12-month threshold) yielded a 52% prevalence and ICD-11 (6-month threshold) yielded a 76% prevalence, a difference that narrowed when both were standardized to a 12-month threshold.

According to the NOS, seven studies demonstrated low risk of bias, while five exhibited higher risks. High-quality studies featured internet-based recruitment, strict age inclusion (≥18 years), and employed validated diagnostic tools, ensuring representativeness and comparability. Conversely, lower-quality studies suffered from high attrition rates (>15%), undermining their reliability. The GRADE framework classified the certainty of evidence as *very low* due to heterogeneity, limited generalizability, and methodological diversity across studies (Dominguez-Rodriguez et al., 2023; Reitsma, Boelen, de Keijser, & Lenferink, 2023; Yu et al., 2024).

Future research should standardize assessment tools, employ randomized designs, and develop AI-driven dynamic scales for real-time symptom monitoring. Integrating biopsychosocial markers via wearable devices and ecological momentary assessment could enhance diagnostic precision, particularly during critical post-loss windows (6–12 months). Establishing multinational cohorts (e.g. WHO grief registry), along with evaluating the long-term efficacy of digital interventions, including machine learning-driven early warning systems and culturally adaptive platforms, could significantly advance evidence-based, personalized care paradigms. Meanwhile, clinical guidelines could evolve to recognize PGD screening as a primary prevention strategy against future dementia and mental health crises. Ultimately, addressing prolonged grief in pandemic-bereaved populations is not only about alleviating current suffering but also about safeguarding the long-term brain health and cognitive resilience of societies recovering from global trauma.

## Conclusion

This systematic review and meta-analysis revealed a high pooled prevalence of PGD observed during the COVID-19 pandemic, indicating substantial geographical heterogeneity that may be potentially linked to cultural coping strategies and differences in the intensity of public health policies. Furthermore, the study underscored the sensitivity of PGD prevalence estimates to the choice of assessment tools and diagnostic criteria. While digital interventions demonstrated promising initial outcomes, their long-term clinical efficacy requires further investigation.

## Supporting information

10.1017/S0033291726104541.sm001 Li et al. supplementary material Li et al. supplementary material

## Data Availability

The data that support the findings of this study are available in the supplementary material of this article.

## Supplementary material

The supplementary material for this article can be found at http://doi.org/10.1017/S0033291726104541.
