# Supplementary material for: Global prevalence of prolonged grief disorder during the COVID-19 pandemic under standardized diagnostic frameworks: A systematic review and meta-analysis
Source: Psychol Med. 2026 May 21;56:e160. doi: 10.1017/S0033291726104541 (PMC13200149; doi:10.1017/S0033291726104541)
Supplement: Li et al. supplementary material [file S0033291726104541sup001.docx]

**Supplementary appendix**

**Supplement to:**

**Global Prevalence of COVID-19-Related Prolonged Grief Disorder Under Standardized Diagnostic Frameworks: A Systematic Review and Meta-Analysis**

**Search Strategy**

**PubMed**

**(**(COVID-19[MeSH Terms]) OR (COVID-19[Title/Abstract]) OR (2019 Novel Coronavirus Disease[Title/Abstract]) OR (2019 Novel Coronavirus Infection[Title/Abstract]) OR (2019-nCoV Disease[Title/Abstract]) OR (2019-nCoV Infection[Title/Abstract]) OR (COVID-19 Pandemic[Title/Abstract]) OR (COVID-19 Pandemics[Title/Abstract]) OR (COVID-19 Virus Disease[Title/Abstract]) OR (COVID-19 Virus Infection[Title/Abstract]) OR (COVID19[Title/Abstract]) OR (Coronavirus Disease 2019[Title/Abstract]) OR (Coronavirus Disease-19[Title/Abstract]) OR (SARS Coronavirus 2 Infection[Title/Abstract]) OR (SARS-CoV-2 Infection[Title/Abstract]) OR (Severe Acute Respiratory Syndrome Coronavirus 2 Infection[Title/Abstract])) AND ((Persistent Complex Bereavement Disorder[Title/Abstract]) OR (Prolonged Grief Disorder[MeSH Terms]) OR (Prolonged Grief Disorder[Title/Abstract]))

**Embase**

('covid-19'/exp OR 'covid-19' OR '2019 novel coronavirus disease'/exp OR '2019 novel coronavirus disease' OR '2019 novel coronavirus infection'/exp OR '2019 novel coronavirus infection' OR '2019-ncov disease'/exp OR '2019-ncov disease' OR '2019-ncov infection'/exp OR '2019-ncov infection' OR 'covid-19 pandemic' OR 'covid-19 pandemics' OR 'covid-19 virus disease' OR 'covid-19 virus infection' OR 'covid19'/exp OR 'covid19' OR 'coronavirus disease 2019'/exp OR 'coronavirus disease 2019' OR 'coronavirus disease-19'/exp OR 'coronavirus disease-19' OR 'sars coronavirus 2 infection'/exp OR 'sars coronavirus 2 infection' OR 'sars-cov-2 infection'/exp OR 'sars-cov-2 infection' OR 'severe acute respiratory syndrome coronavirus 2 infection'/exp OR 'severe acute respiratory syndrome coronavirus 2 infection') AND ('prolonged grief disorder'/exp OR 'prolonged grief disorder' OR 'persistent complex bereavement disorder'/exp OR 'persistent complex bereavement disorder')

**
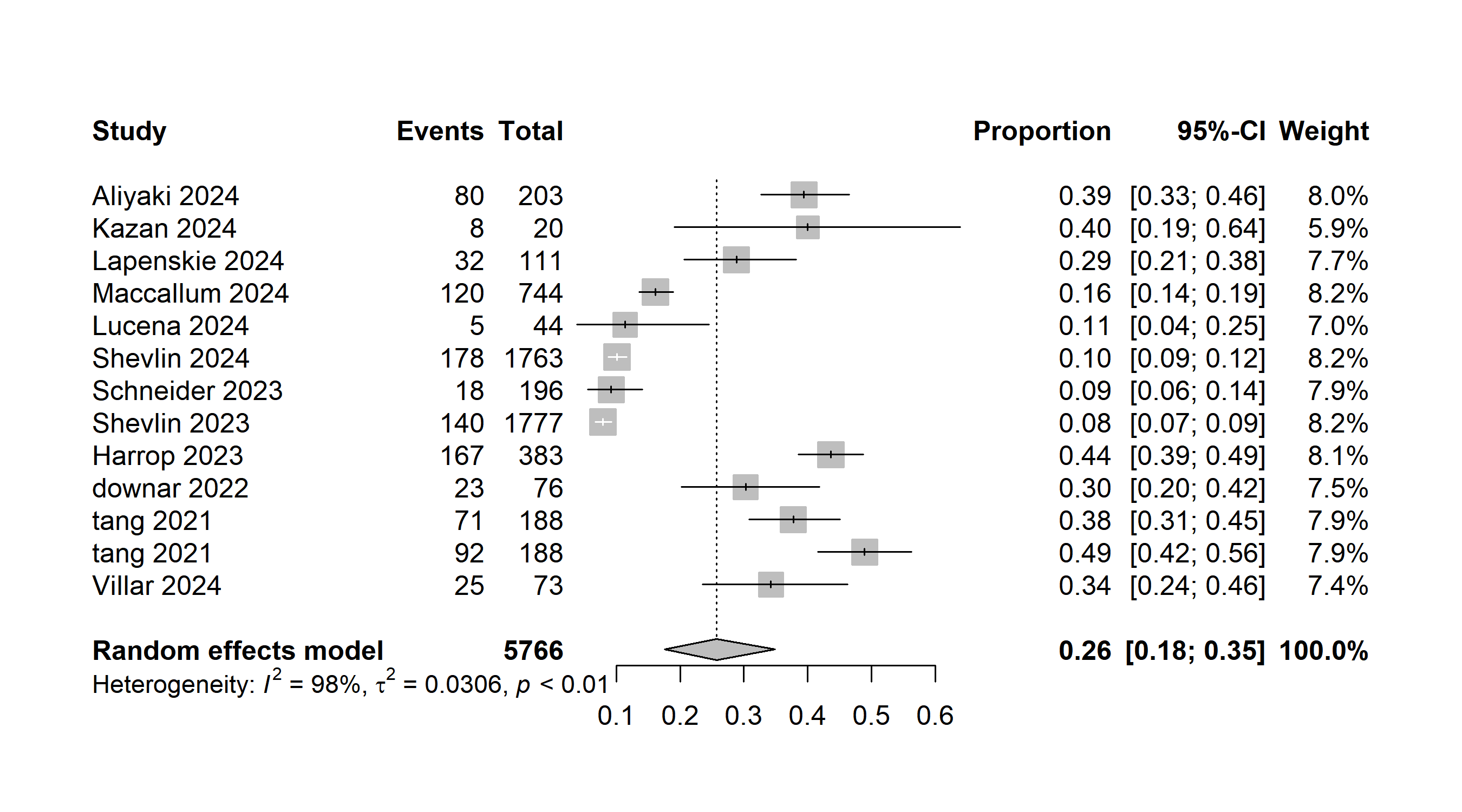
**

**Figure S1. Pooled Meta-Analysis Prevalence Rates for COVID-19-Related PGD in Lenient Criteria.**


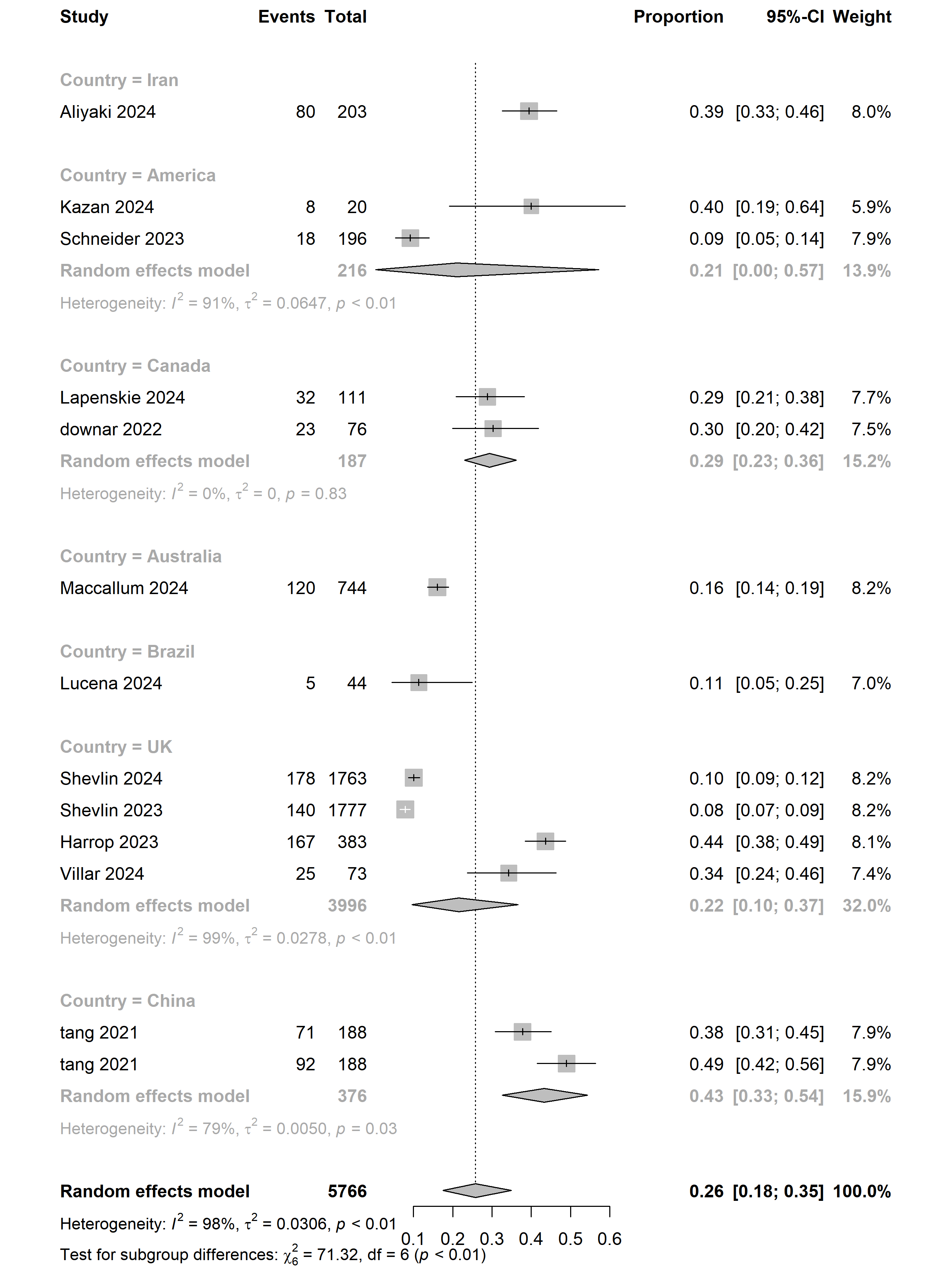


**Figure S2. Pooled Meta-Analysis Prevalence Rates for COVID-19-Related PGD in National Subgroups Under Lenient Criteria.**


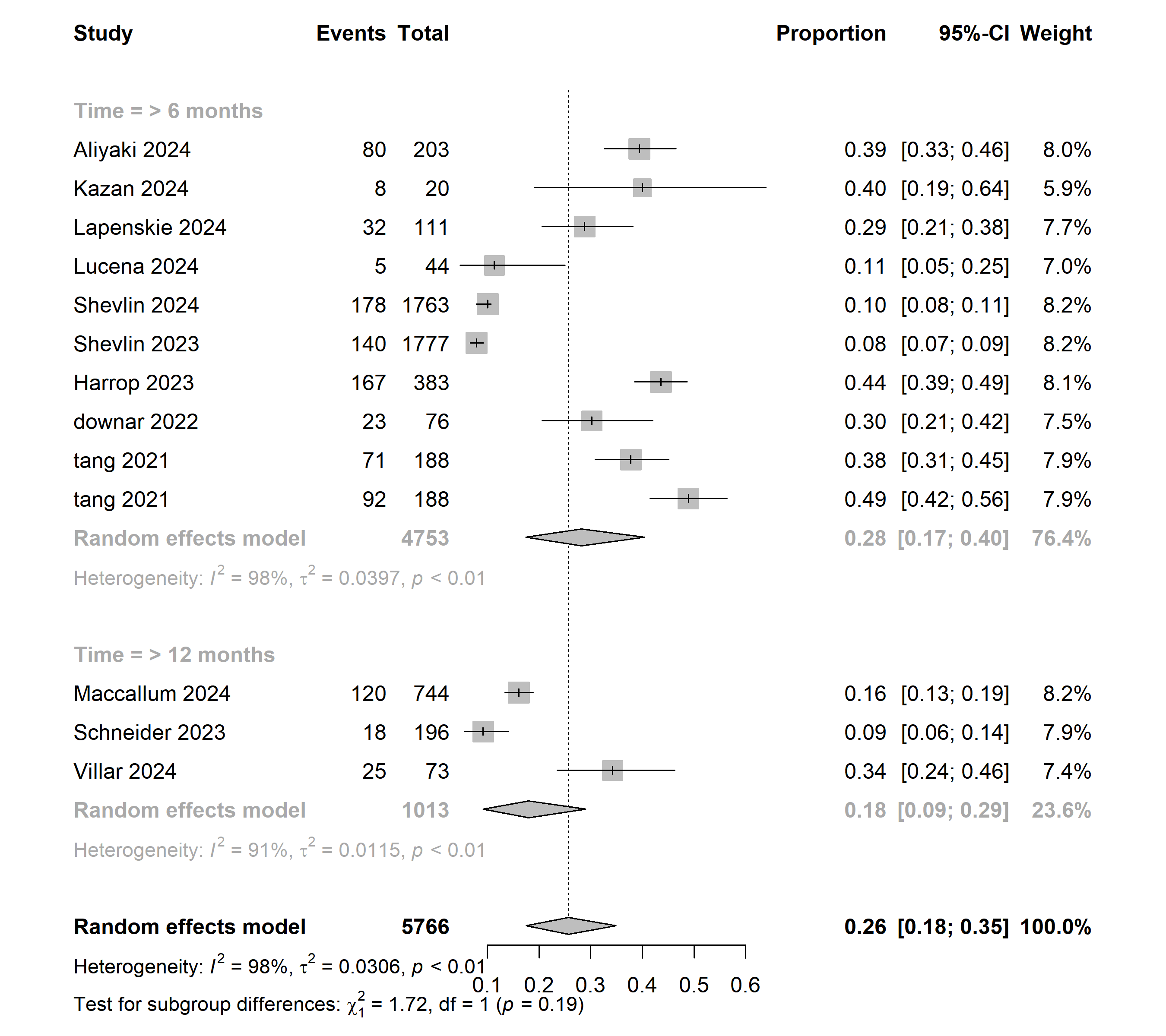


**Figure S3. Pooled Meta-Analysis Prevalence Rates for COVID-19-Related PGD in Follow-up Time Subgroups Under Lenient Criteria.**


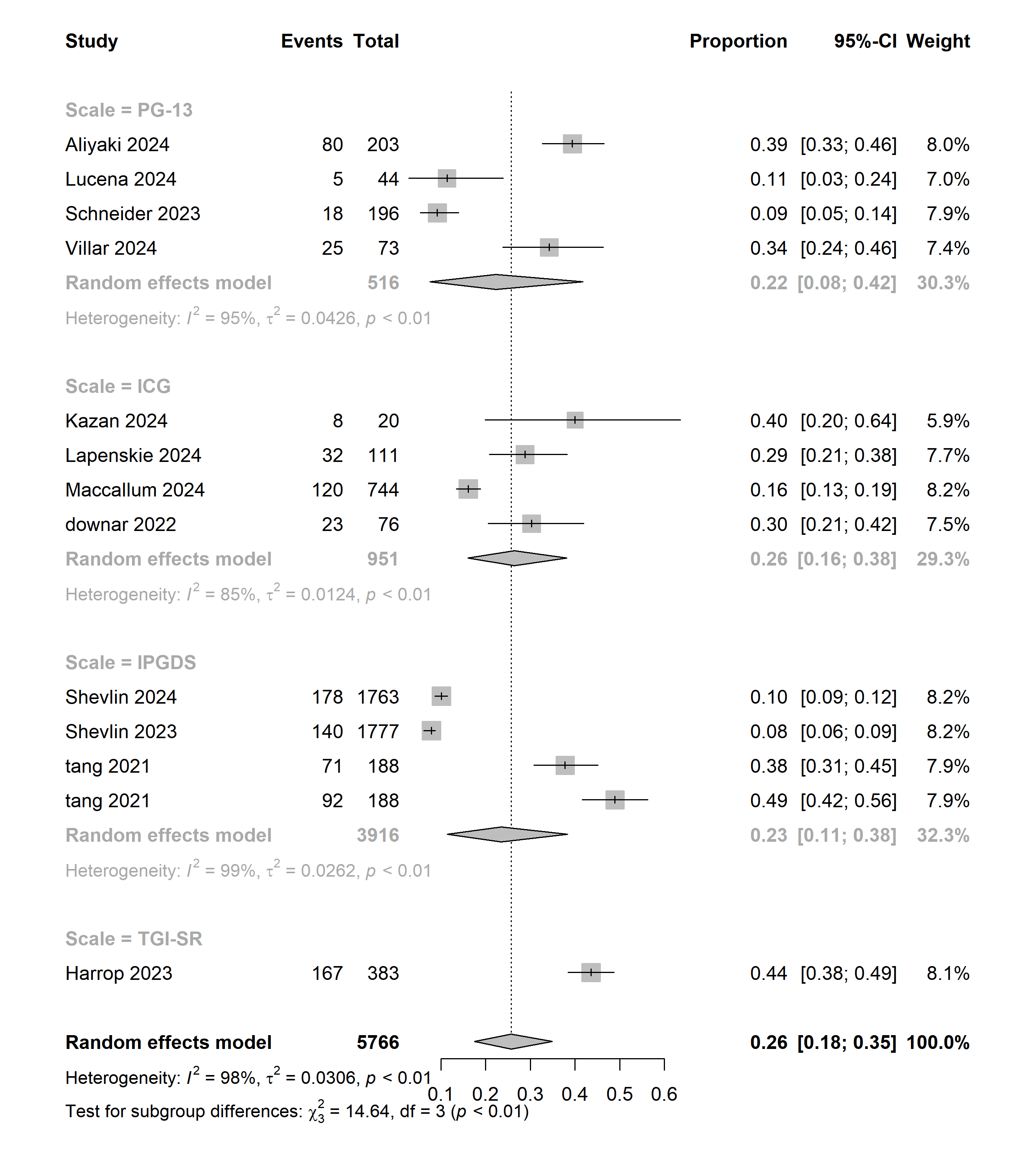


**Figure S4. Pooled Meta-Analysis Prevalence Rates for COVID-19-Related PGD in Scale Subgroups Under Lenient Criteria.**


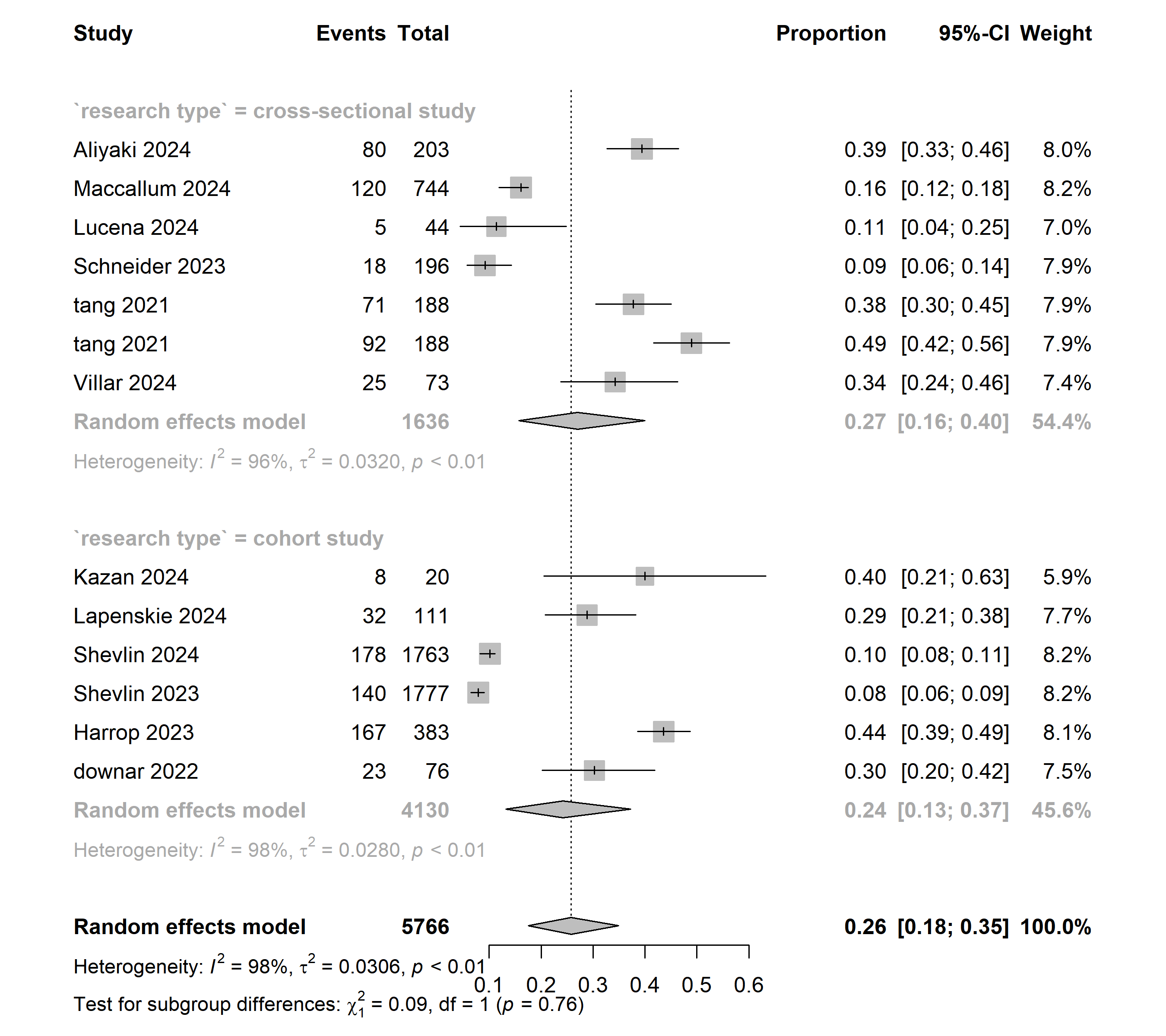


**Figure S5. Pooled Meta-Analysis Prevalence Rates for COVID-19-Related PGD in Study Design Subgroups Under Lenient Criteria.**

**
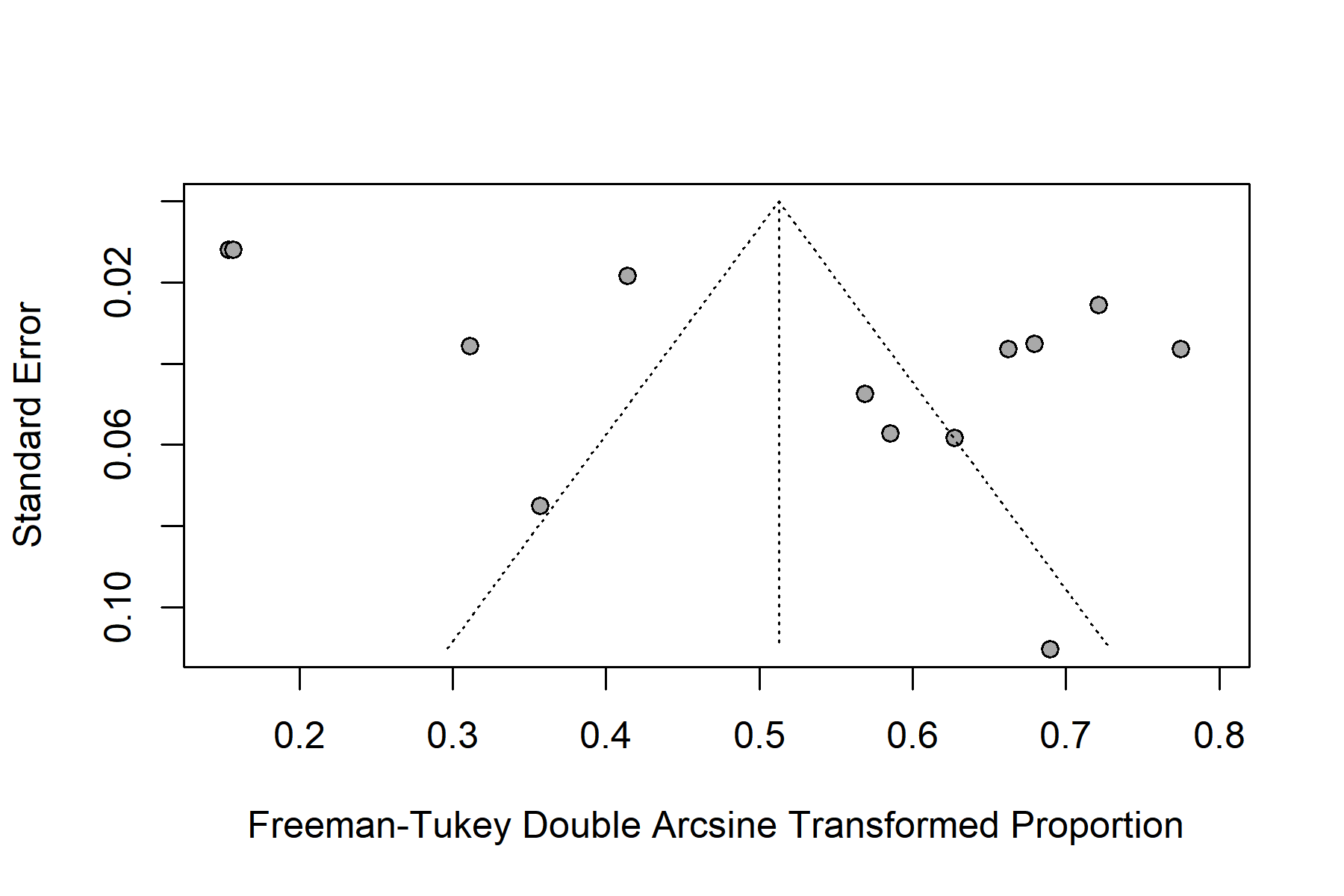
**

**Figure S6. Funnel Plot of Results for COVID-19-related PGDs under Strict Criteria.**

**
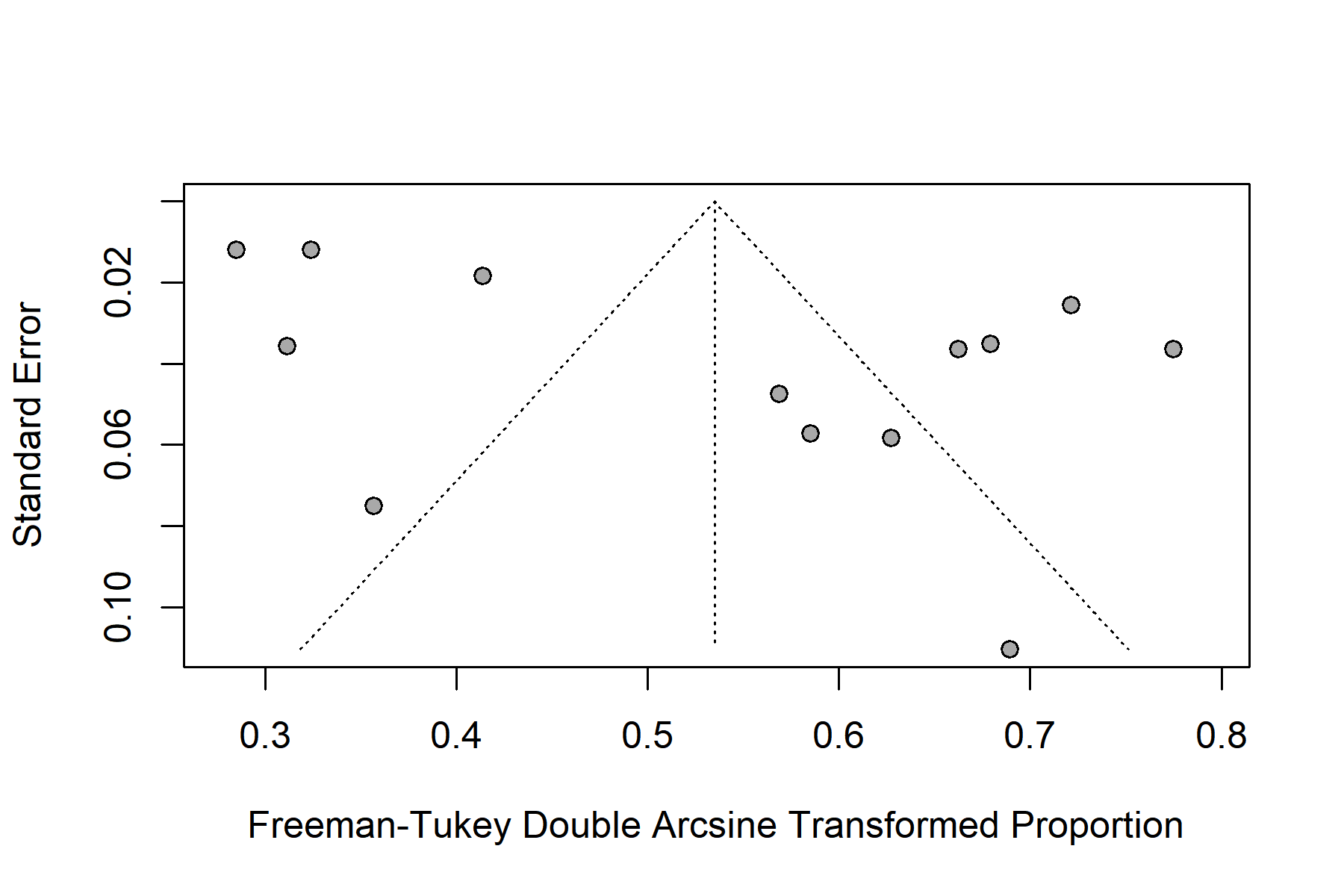
**

**Figure S7. Funnel Plot of Results for COVID-19-related PGDs under Lenient Criteria.**

**
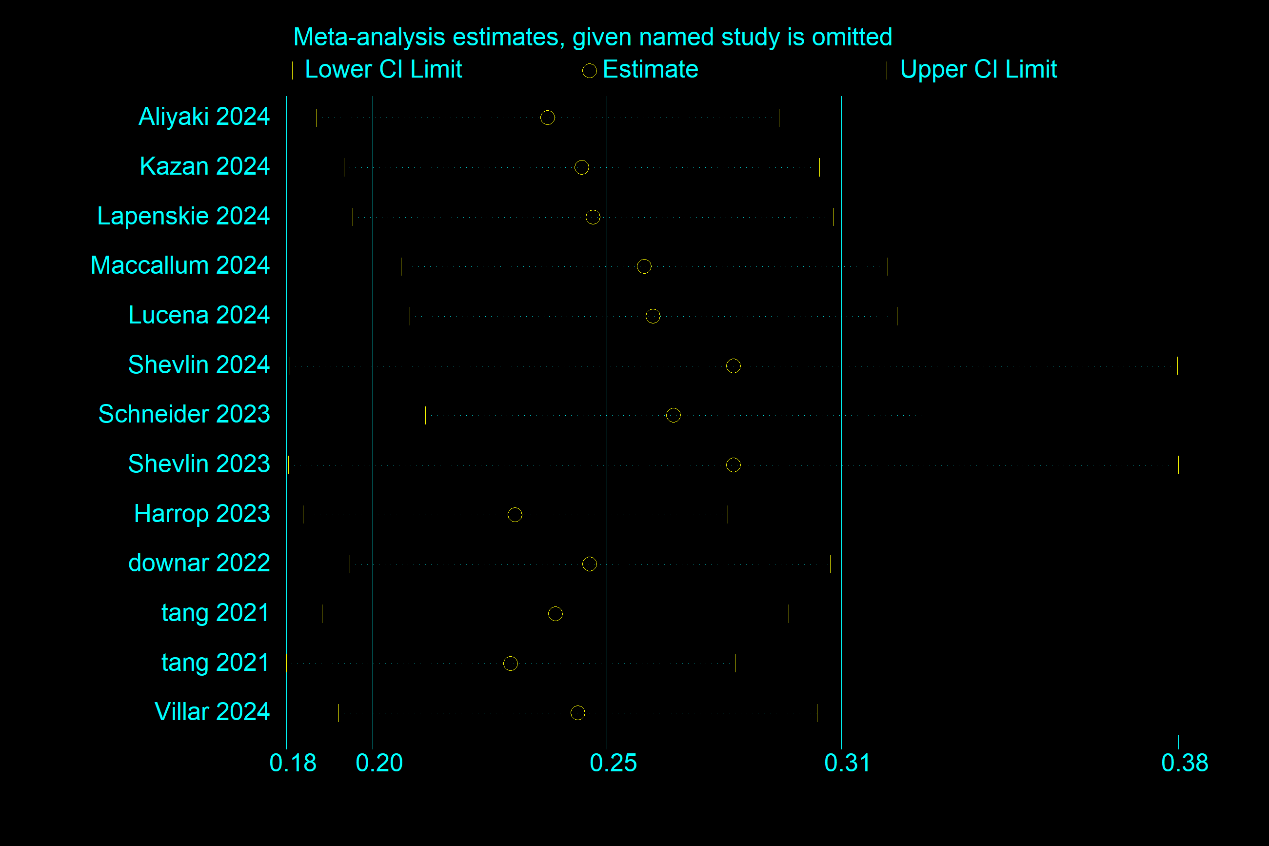
**

**Figure S8. Sensitivity Analysis of Results for COVID-19-related PGDs.**


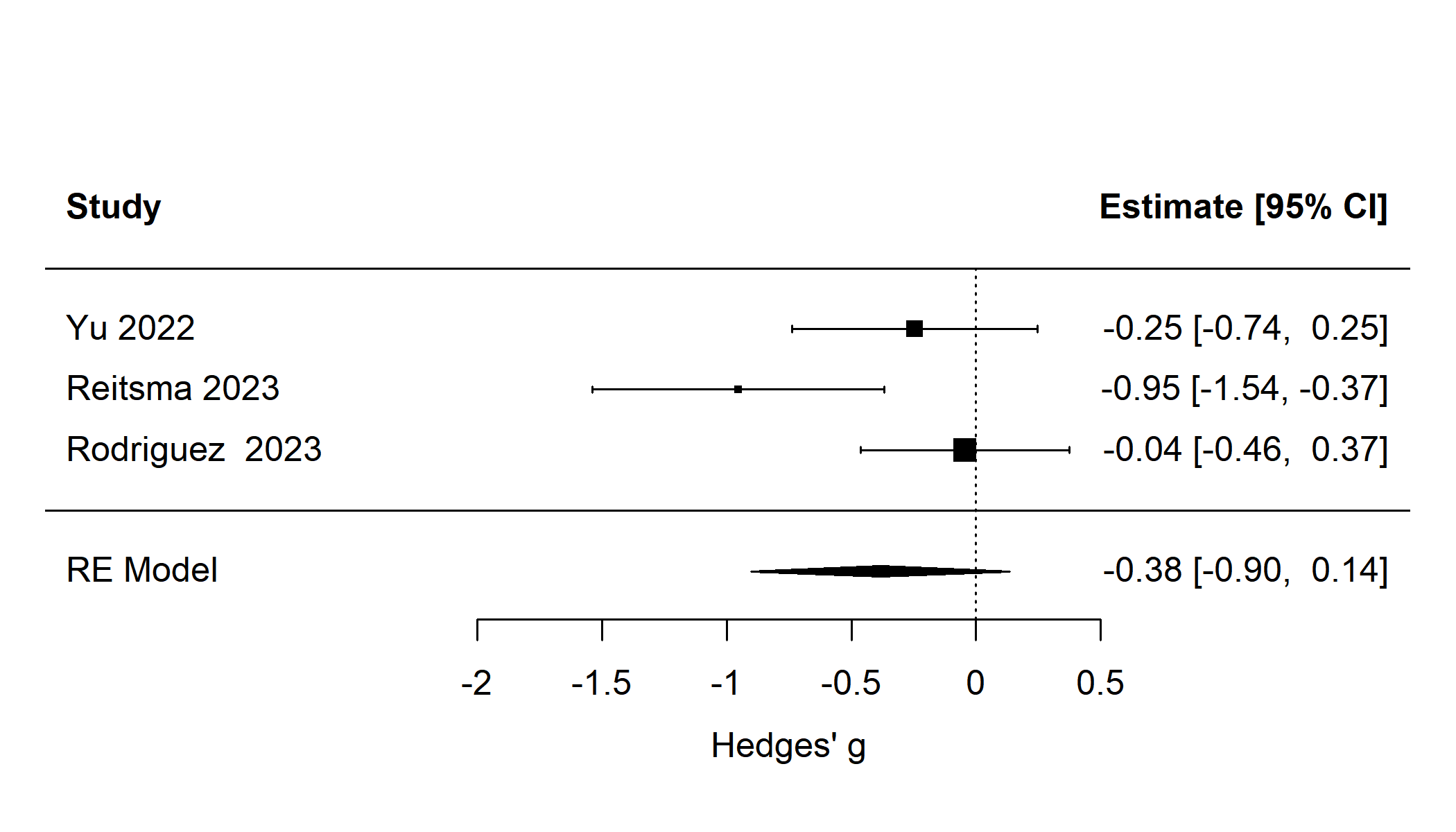


**Figure S9. Results on Efficacy of Digital PGD Interventions**

**Table S1. Risk of bias assessments according to the Newcastle-Ottawa Scale.**

| **No.** | **Author (year)** | **Study design** | **Selection** | **Comparability** | **Exposure/Outcome** | **Total** |
| --- | --- | --- | --- | --- | --- | --- |
| 1 | Aliyaki 2024 | cross-sectional study | 4 | 2 | 2 | 8 |
| 2 | Kazan 2024 | cohort study | 3 | 2 | 2 | 7 |
| 3 | Lapenskie 2024 | cohort study | 4 | 2 | 3 | 9 |
| 4 | Maccallum 2024 | cross-sectional study | 4 | 2 | 2 | 8 |
| 5 | Lucena 2024 | cross-sectional study | 4 | 2 | 3 | 9 |
| 6 | Shevlin 2024 | cohort study | 4 | 2 | 3 | 9 |
| 7 | Schneider 2023 | cross-sectional study | 3 | 2 | 3 | 8 |
| 8 | Shevlin 2023 | cohort study | 4 | 2 | 2 | 8 |
| 9 | Harrop 2023 | cohort study | 4 | 2 | 3 | 9 |
| 10 | downar 2022 | cohort study | 4 | 2 | 3 | 9 |
| 11 | Tang 2021 | cross-sectional study | 4 | 2 | 3 | 9 |
| 12 | Tang 2021 | cross-sectional study | 4 | 2 | 3 | 9 |
| 13 | Rodriguez-Villar 2024 | cross-sectional study | 4 | 2 | 3 | 9 |
| 14 | Yu 2022 | cohort study | 3 | 1 | 2 | 6 |
